# Microbiology Profile of COVID-19-Associated Rhino-Orbital Mucormycosis Pathogens in South India

**DOI:** 10.4269/ajtmh.22-0411

**Published:** 2022-12-26

**Authors:** Esther Sheba, Savitri Sharma, Dilip Kumar Mishra, Tarjani Vivek Dave, Anasua Ganguly Kapoor, Joveeta Joseph

**Affiliations:** ^1^ Jhaveri Microbiology Centre, Brien Holden Eye Research Centre, L V Prasad Eye Institute, Hyderabad, India;; ^2^ The Ramoji Foundation for Ocular Infections, L V Prasad Eye Institute, Hyderabad, India;; ^3^ Ophthalmic Pathology Laboratory, L V Prasad Eye Institute, Hyderabad, India;; ^4^ Ophthalmic Plastic Surgery Service, L V Prasad Eye Institute, Hyderabad, India;; ^5^ Ophthalmic Plastic and Facial Aesthetic, Orbit and Ocular Oncology, L V Prasad Eye Institute, Vijayawada, India

## Abstract

This study describes the microbiological and histopathological features of patients with COVID-19-associated rhino-orbital mucormycosis (ROM) seen at the L V Prasad Eye Institute between May and August 2021. Diagnosed clinically and radiologically, 24 patients with ROM were included in the study. Deep nasal swabs or endoscopically collected nasal swabs or orbital tissues were submitted for microbiological evaluation and in vitro susceptibility testing by microbroth dilution for natamycin, amphotericin B, caspofungin, posaconazole, ketoconazole, and voriconazole. Cultures were processed by 28S ribosomal DNA polymerase chain reaction and molecular sequencing. A portion of orbital tissues was also sent for histopathological evaluation. The age of the patients ranged from 27 to 75 (mean 48.58 ± 14.09) years and the majority (79%) were male. Nineteen patients were known to be diabetic prior to developing ROM and 18 patients had recovered from active COVID-19 infection. Thirteen patients had a history of hospitalization during COVID-19 infection and eight received steroids. Of the 24 samples, microbiological evaluation identified *Rhizopus arrhizus* in 12, *Rhizopus microsporus* in 9, *Lichtheimia ramosa* in 2, and *Rhizopus delemar* in 1. Twelve isolates were tested for antifungal susceptibility and all were susceptible to natamycin and amphotericin B. The susceptibility to posaconazole was high, with minimum inhibitory concentration (MIC) < 2 µg/mL for 10/12 (84%) isolates, whereas the MIC of other drugs varied. Histopathological examination of tissues showed acute fulminant disease, granuloma formation, and vascular invasion by the fungal pathogens in these specimens. *Rhizopus arrhizus* was predominantly associated with ROM and most isolates were susceptible to amphotericin B and posaconazole. Further studies are needed to corroborate the findings and explain possible underlying links.

## INTRODUCTION

Rhino-orbital mucormycosis (ROM) is a rare fulminant fungal infection recognized primarily in immunocompromised people or those with preexisting comorbidities and underlying pathology.^1^ However, during the second wave of COVID-19 due to the delta variant, an exponential rise in these infections was seen worldwide and India contributed to a major proportion. Mucormycosis was then declared an epidemic in India among patients with COVID-19 in May 2021.^1^^,^^2^ The COVID-19 pandemic, along with prolonged hospital stay and immunosuppressive therapy, especially steroids, in association with diabetes, and irrational use of antibiotics, helped the propagation of fungal pathogens in other ocular infections as well.^3^^,^^4^ However, most evident was mucormycosis, which involved the sinuses, orbits, and brain.^1^^,^^2^ The infiltrating fungus destroys the surrounding bone and soft tissue through vascular thrombosis and subsequent tissue infarction and may reach the brain with fatal complications.^5^ Because ROM is a rapidly progressive disease,^6^^,^^7^ any delay in appropriate management can have a serious adverse effect on patient survival. Empiric treatment with intravenous liposomal amphotericin B and surgical debridement of nose and paranasal sinus to reduce infective tissue is the mainstay, although prognosis is poor. In refractory cases or patients intolerant to amphotericin B, posaconazole is considered an alternative or add-on therapy, and increasing the bioavailability of the drug intraorbital amphotericin B has also been tried with varied results.^8^ A recently published large multicentric study from India—COSMIC Report 1—has studied the association of COVID-19 and rhino-orbito-cerebral mucormycosis (ROCM) in depth and determined risk factors as well as clinical profiles.^9^ The primary result showed that diabetes was present in 78% ofz patients, whereas 87% had been treated with steroids. Successful outcomes depended on a high index of clinical suspicion, prompt diagnosis, early treatment with amphotericin B, aggressive surgical debridement of the paranasal sinus, and orbital exenteration where indicated.^9^

*Mucor* is a fungus of member Zygomycetes, order Mucorale,^10^ whose spores are normally present in the environment. Following *Aspergillus*, Mucorales fungi are the next most common pathogens in immunocompromised individuals. Despite being termed an “epidemic,” the incidence of mucormycosis was underestimated, partly due to the rarity of the condition, and partly to unavailability of confirmatory tissue biopsy for diagnosis.^5^ Diagnosis is based on early clinical suspicion, computational tomography of paranasal sinus, orbit, and brain, and preliminary microbiological magnetic resonance imaging in cases of suspected intracranial spread and microbiological culture or biopsy.^11^ There is a paucity of data regarding antifungal susceptibility and species identification of this group of pathogens that has affected our country. Therefore, we performed this study to analyze the microbiological and histopathological features of COVID-19-associated ROCM.

## MATERIALS AND METHODS

All consecutive confirmed cases of mucormycosis (*N* = 24) that were diagnosed clinically and radiologically along with demonstration of the fungus in tissue (broad, aseptate, or pauciseptate hyphae with wide-angle branching and evidence of tissue invasion) via direct microscopy/culture/histopathological examination (HPE) at the L V Prasad Eye Institute between May and July 2021 were included in the study. The subjects were thoroughly evaluated with detailed history taking and ophthalmological, throat, and sinus examinations along with imaging studies. Debridement of local necrotic tissue through endoscopic or open approaches or orbital floor clearance was done where indicated. Exenteration was also performed when there was documented progression of disease despite maximal medical therapy and surgical debridement. Deep nasal swabs or endoscopically collected nasal swabs or orbital mass tissues were submitted to the microbiology laboratory and, whenever available, a portion of the biopsy was sent for histopathological processing as well.

### Microbiological processing.

Smears were made on glass slides stained with 10% potassium hydroxide with 0.1% calcofluor white (KOH + CFW) and examined by fluorescence microscopy for the presence of fungal filaments suggestive of Mucorales. The samples were also inoculated onto chocolate (5% sheep blood) agar and potato dextrose agar. The former was incubated at 37°C and the latter at 27°C for 1–2 weeks. Mucorales grown were identified based on hyphal and spore morphology by lactophenol cotton blue staining as well as using molecular techniques. Whenever possible, cultures were also processed for antifungal susceptibility testing by the microbroth dilution method described below.

### Antifungal susceptibility testing.

Antifungal susceptibility testing was done for 12 out of 24 isolates as per Clinical Laboratory Standard Institute guidelines (CLSI M38-A2, 2008) against six drugs, namely amphotericin B (A9528), natamycin (32417), caspofungin (SML0425), posaconazole (32103), voriconazole (PZ0005), and ketoconazole (K10003) procured from Sigma-Aldrich (St. Louis, MO). The remaining isolates could not be tested because they were mixed with *Aspergillus* sp. and pure colonies could not be isolated. The drugs were reconstituted in dimethyl sulfoxide and diluted to a drug concentration of 1,600 µg/mL for amphotericin, posaconazole, voriconazole, and ketoconazole, 800 µg/mL for caspofungin, and 3,200 µg/mL for natamycin. The drugs were further diluted to a working concentration ranging from 32 to 0.06 µg/mL for amphotericin B, posaconazole, ketoconazole, and voriconazole, 64 to 0.12 µg/mL for natamycin, and 16 to 0.01 µg/mL for caspofungin, and were prepared in sterile RPMI-1640 medium with l-glutamine, phenol red, and 0.2% glucose without sodium bicarbonate (HiMedia, Mumbai, India). The fungal suspension was made by gently teasing the spores from the agar surface with phosphate-buffered saline. The suspension was transferred to a sterile tube and density was adjusted to 0.5 McFarland optical density using a densitometer (DensiCHEK Plus, bioMerieus, India). The suspension was further diluted to 1:50 in RPMI medium to obtain a standard inoculum of 0.4–5.0 × 10^4^ colony-forming units per milliliter. On a microtiter plate, 100 μL of each drug dilution and 100 μL of the diluted fungal inoculum were added to all the wells and incubated for 48 hours at 27°C. The plates were observed visually for the presence of growth and the lowest concentration at which the fungal growth was completely inhibited was recorded as the minimum inhibitory concentration (MIC) value. *Aspergillus flavus* (ATCC 9643) was used for standardization of the test and appropriate controls were included. Susceptibility was based on CLSI guidelines (M38-A2)^12^ for all the antifungal agents, except natamycin, for which the susceptibility breakpoint was based on published literature.^13^

### Molecular identification by polymerase chain reaction.

The fungal mat from the culture was chopped into fine pieces in a sterile Petri dish and transferred to a 1.5-mL microcentrifuge tube. Genomic DNA was extracted using a NucleoSpin Plant II kit (740770.250, Macherey-Nagel) as per the manufacturer’s instructions. Polymerase chain reaction (PCR) was set up using DreamTaq Green PCR Master Mix (2×) (K1081, Thermo Fisher Scientific), along with 28S ribosomal RNA (rRNA) primers (forward primer: 5′-GTGAAATTGTTGAAAGGGAA-3′; reverse primer: 5′-GACTCCTTGGTCCGTGTT-3') as described earlier.^14^ Polymerase chain reaction amplification was carried out on a C1000 Touch PCR thermal cycler (Bio-Rad Technologies) under conditions of initial denaturation at 94°C for 4 minutes followed by 34 cycles of denaturation at 94°C for 30 seconds, annealing at 58°C for 1 minute, extension at 72°C of 1 minute, and final extension at 72°C for 1 minute. The PCR products were subjected to agarose gel electrophoresis with 1% agarose gel and the amplicons were purified using a QIAquick PCR purification kit (28106, Qiagen) according to the manufacturer’s protocol and sequenced using BigDye Terminator v. 3.1 (Applied Biosystems). The sequences were then subjected to BLAST search for species identification and submitted to the NCBI database (https://blast.ncbi.nlm.nih.gov/Blast.cgi), and accession numbers were obtained (Table 1).

**Table 1:** Clinical and demographic features of the patients included in the ROM study

Sample number	Age (years)	Sex	History of COVID-19	Diabetic	History of steroids	Management	Visual acuity	Specimen
1	40	F	No	Yes	No	TRAMB	20/40	Pus
2	73	M	Yes	Yes	Yes	TRAMB	No vision	Nasal swab
3	60	F	Yes	No	Yes	Exenteration	No vision	Necrotic material
4	41	M	Yes	Yes	Yes	Exenteration	20/100	Orbital abscess
5	33	M	Yes	Yes	Yes	Exenteration	20/20	Swab
6	65	F	Yes	No	Yes	Exenteration	No vision	Necrotic material
7	60	M	Yes	Yes	Not known	Exenteration	NPL	Swab
8	57	F	No	Yes	No	TRAMB	NPL	Deep nasal swab
9	44	M	Yes	Yes	Yes	TRAMB	20/20	Orbital abscess
10	27	M	No	Yes	No	TRAMB	NPL	Endoscopy nasal swab
11	75	M	Yes	Yes	No	TRAMB	NPL	Nasal swab
12	38	M	Yes	Yes	Yes	Exenteration	NPL	Orbital mass tissue
13	45	M	No	Yes	Yes	TRAMB + orbital debridement	20/50	Right nasal swab
14	33	M	Yes	Yes	No	Exenteration	NPL	Nasal biopsy
15	55	F	No	No	Not known	TRAMB + orbital debridement	20/125	Endoscopy nasal swab
16	48	M	No	Yes	Yes	TRAMB + orbital debridement	NPL	Endoscopy nasal swab
17	60	M	Yes	Yes	Not known	TRAMB + orbital debridement	NPL	Apical mass
18	45	M	Yes	Yes	Yes	TRAMB + orbital debridement	NPL	Orbital mass tissue
19	71	M	Yes	Yes	Yes	TRAMB + orbital debridement	NPL	Nasal swab
20	35	M	Yes	Yes	No	TRAMB + exenteration	PL + PR	Orbital mass tissue
21	43	M	No	No	No	TRAMB + exenteration	NPL	Orbital mass
22	40	M	Yes	No	No	Exenteration	NPL	Excised tissue
23	51	M	Yes	Yes	Yes	TRAMB + orbital debridement	NPL	Orbital mass tissue
24	27	M	Yes	Yes	Yes	TRAMB + orbital debridement	NPL	Orbital tissue

NPL = no light perception; PL+PR = accurate perception of light and rays; ROM = rhino-orbital mucormycosis; TRAMB = transcutaneous retrobulbar amphotericin B.

### Histopathological processing.

Paraffin-embedded sections of tissue samples were deparaffinized using serial solutions of xylene (35417, Thermo Fisher Scientific, Mumbai, India) and hydrated through serial dilutions of 100%, 90%, and 80% alcohol (26897, Thermo Fisher Scientific). They were further washed and oxidized in periodic acid-Schiff (PAS) (375810-25G, Sigma-Aldrich, China) solution for 5 minutes. After rinsing, the tissue sections were placed in Coleman’s Schiff reagent (857343, Merck, India) for 15 minutes and counterstained with Harris hematoxylin solution for 15 min and again washed. Dehydration was done with 80%, 90%, and 100% alcohol, cleared with xylene, and mounted with DPX mount medium (46029, Fine-Chem, Mumbai, India). Additionally, paraffin sections of tissue samples were also oxidized with 4% chromic acid (37762KO5, SDFCL, Mumbai, India) solution for 1 hour and treated with 1% sodium bisulfate (05796, Loba Chemie, Mumbai, India) for 1 minute. The chemically treated tissue was heated in a working solution of silver nitrate (S8157-25G, Sigma-Aldrich, India) and methenamine (3843KO5, SDFCL) until the section became golden brown in color. This section was rinsed in running tap water for a few minutes and treated with 0.1% gold chloride (Sigma-Aldrich, India) solution for 1 minute followed by 2% sodium thiosulfate (40235KO5, SDFCL) and counterstained with 1% Light Green (Fine-Chemicals, India). Dehydration was done with 80%, 90%, and 100% alcohol, cleared with xylene, and mounted with DPX mount medium (25832, Sigma-Aldrich, India).

## RESULTS

Over the study period, a total of 24 patients were diagnosed with ROM based on microbiological and/or HPE of the clinical samples. Demographic and clinical features of the patients are presented in Table 1. Of the total patients, 19 were males and 5 were females and the age range of the patients was between 27 and 75 years. The most commonly affected age group was 36–45 years, and the mean age of the patients was 48.5 ± 14.09 years. Eighteen of the 24 patients developed ROM following active COVID-19 infection, whereas six patients did not have a history of known active COVID-19 infection. A large number of patients (*N* = 19) had a history of type II diabetes mellitus and 13 patients received systemic corticosteroids during the course of their treatment of COVID-19. Seventeen eyes (70.8%) had no perception of light at presentation whereas one patient had perception of light only. Six patients had a presenting visual acuity of < 20/125. All the patients were treated with transcutaneous retrobulbar amphotericin B (TRAMB) and systemic liposomal amphotericin B. Ten eyes (41.6%) underwent exenteration whereas eight eyes had debridement of the necrotic sinonasal tissue. A favorable final outcome with respect to management of ROM was seen in 18 (75%) cases.

**Figure 1.**
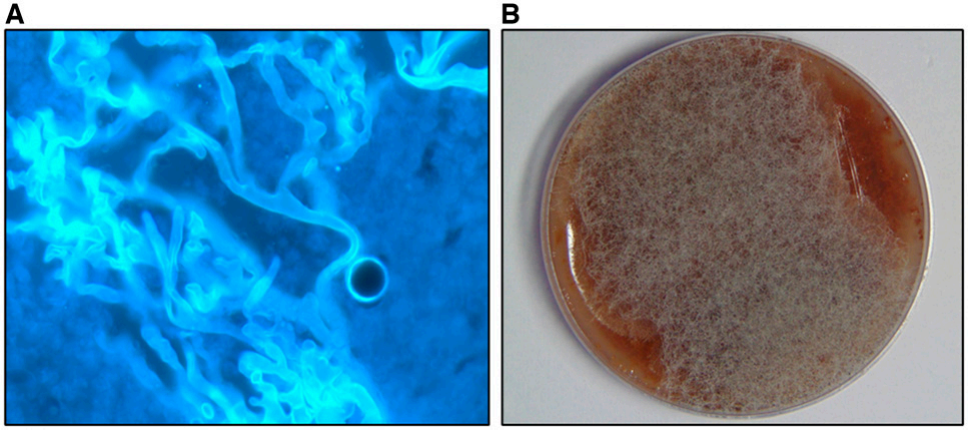
Microscopic and culture identification of Mucorales. Orbital tissues with presumed mucormycosis were subjected to routine microbiological workup. (**A**) Microscopy (40×) image of KOH + calcofluor white showing aseptate broad ribbon-like filaments. (**B**) Fungal growth on chocolate agar media exhibiting cottony white colonies that turned brown, gray, or black with sporulation.

Microscopic examination confirmed the presence of fungal filaments, which were aseptate or sparsely septate with broad thin-walled or irregular ribbon-like hyphae in 17/24 samples, as shown in Figure 1A. On culture media the colonies appeared cottony white, which turned brown, gray, or black with the time corresponding to sporulation (Figure 1B). Culture was positive for Mucorales in 24/24 samples and was predominantly identified as *Rhizopus arrhizus* (21/24) by lactophenol cotton blue, which showed hyaline broad aseptate hyphae, brown sporangiophores, sporangia, and sporangiospores and rhizoids at the base. All the Mucorales grown were further amplified using the 28S rRNA large subunit, which corresponds to an amplicon size of 340 bp. The amplified DNA was subjected to Sanger sequencing and the results identified the following Mucorales: 11/24 isolates were identified as *R. arrhizus*, 9/24 as *Rhizopus microsporus*, whereas 2 were identified as *Lichtheimia ramosa*, 1 as *Rhizopus homothallicus*, and 1 as *Rhizopus delemar* (Table 2). The identified sequences have been deposited in the NCBI GenBank database with the accession numbers shown in Table 2. Among the 10 eyes that progressed despite medical therapy and surgical debridement, *R. arrhizus* was isolated in five eyes and *R. microsporus *in four, whereas one eye showed the presence of *R. delemar*. Similarly, five eyes with *R. arrhizus* and three eyes infected with *R. microsporus* underwent surgical debridement along with TRAMB. There was no relevance of the identity of the fungal species to the plan of management and outcome.

**Table 2:** Microbiological details of identification of the 24 isolates of Mucorales by culture and 28S rDNA and their GenBank accession numbers

Sample number	Specimen	Direct microscopy	Morphological identification by LPCB	Identification by DNA sequencing	Accession number
1	Pus	Broad, aseptate, hyaline, bulbous fungal filaments	*Rhizopus arrhizus*	*R. arrhizus*	OM843124
2	Nasal swab	No organisms	Unidentified Mucorales	*Lichtheimia ramosa*	OM971062
3	Necrotic material	Aseptate, broad, hyaline fungal filaments with ribbon-like folds	*R. arrhizus*	*R. arrhizus*	OM843118
4	Orbital abscess	Thin, septate fungal filament	*R. arrhizus*	*Rhizopus microsporus*	OM971063
5	Nasal swab	Aseptate, broad, hyaline fungal fragment	*R. arrhizus*	*R. arrhizus*	OM843121
6	Necrotic material	One broad, aseptate, fungal fragment	*R. arrhizus*	*R. arrhizus*	OM843119
7	Nasal swab	Aseptate, broad, hyaline fungal filaments with ribbon-like folds	*R. arrhizus*	*R. microsporus*	OM859002
8	Nasal swab	Aseptate, broad, hyaline fungal filaments with ribbon-like folds	*R. arrhizus*	*R. microsporus*	OM859007
9	Orbital abscess	Broad, aseptate, hyaline fungal filaments	*Rhizopus azygosporus*	*Rhizopus homothallicus*	OM964570
10	Nasal swab	Budding yeast cells	*Mucor *species	*L. ramosa*	OM971064
11	Nasal swab	Budding yeast with pseudohyphae	*R. arrhizus*	*R. microsporus*	OM842971
12	Orbital mass tissue	Aseptate, broad, hyaline fungal filaments	*R. arrhizus*	*R. arrhizus*	OM843116
13	Nasal swab	Aseptate, broad, hyaline fungal filaments	*R. arrhizus*	*R. arrhizus*	OM843117
14	Nasal biopsy	Aseptate, broad, hyaline fungal filaments with ribbon-like folds	*R. arrhizus*	*R. arrhizus*	OM843120
15	Nasal swab	Aseptate, broad, hyaline fungal filaments with ribbon-like folds	*R. arrhizus*	*R. microsporus*	OM859003
16	Endonasal swab	No organisms	*R. arrhizus*	*R. microsporus*	OM859004
17	Apical mass	Hazy, broad fungal filaments	*R. arrhizus*	*R. microsporus*	OM859005
18	Orbital tissue	Broad, aseptate fungal filaments	*R. arrhizus*	*R. arrhizus*	OM843122
19	Nasal swab	Broad, aseptate fungal filaments with ribbon-like folds	*R. arrhizus*	*R. arrhizus*	OM843123
20	Orbital tissue	Broad, aseptate fungal filaments	*R. arrhizus*	*R. microsporus*	OM859006
21	Orbital mass	–	*R. arrhizus*	*Rhizopus delemar*	OM859008
22	Excised tissue	Broad, aseptate nonfluorescing filaments with ribbon-like folds	*R. arrhizus*	*R. microsporus*	OM964569
23	Orbital mass tissue	Broad, aseptate hyaline fungal filaments with ribbon-like folds	*R. arrhizus*	*R. arrhizus*	OM964572
24	Orbital tissue	No organisms	*R. arrhizus*	*R. arrhizus*	OM964571

LPCB = lactophenol cotton blue wet mount.

### Antifungal susceptibility testing.

Minimum inhibitory concentration testing was performed for 12/24 isolates using a microbroth dilution method and the results are shown in Table 3. Ten of the 12 *Mucorales* spp. were susceptible to posaconazole with an MIC < 2 µg/mL as well as natamycin, wherein the MIC was < 8 µg/mL for all the isolates, depicting moderate susceptibility. However, the MIC for voriconazole, ketoconazole, and caspofungin was variable from 2 to 8 µg/mL and indicated a high degree of resistance. Only amphotericin B and posaconazole had good susceptibility in vitro.

**Table 3:** Antifungal susceptibility testing of 12/24 isolates that could be isolated in pure culture from patients with ROM

Sample number	Culture identification	MIC amphotericin B (µg/mL)	MIC voriconazole (µg/mL)	MIC ketoconazole (µg/mL)	MIC natamycin (µg/mL)	MIC posaconazole (µg/mL)	MIC caspofungin (µg/mL)
1	*Rhizopus arrhizus*	4	16	4	8	0.5	> 8
2	Unidentified Mucorales	2	1	16	2	2	> 8
3	*R. arrhizus*	4	16	> 16	8	1	> 8
4	*R. arrhizus*	4	4	> 16	4	2	> 8
5	*R. arrhizus*	2	16	8	8	1	> 8
6	*R. arrhizus*	4	16	8	8	2	> 8
7	*R. arrhizus*	4	4	8	8	2	> 8
8	*R. arrhizus*	4	16	16	8	2	> 8
9	*Rhizopus azygosporus*	2	8	8	8	2	> 8
10	*Mucor *species	2	16	0.5	8	0.125	> 8
11	*R. arrhizus*	4	2	16	2	> 16	2
12	*R. arrhizus*	4	2	16	2	> 16	2

MIC = minimum inhibitory concentration; ROM = rhino-orbital mucormycosis.

### Histopathological findings.

**Figure 2.**
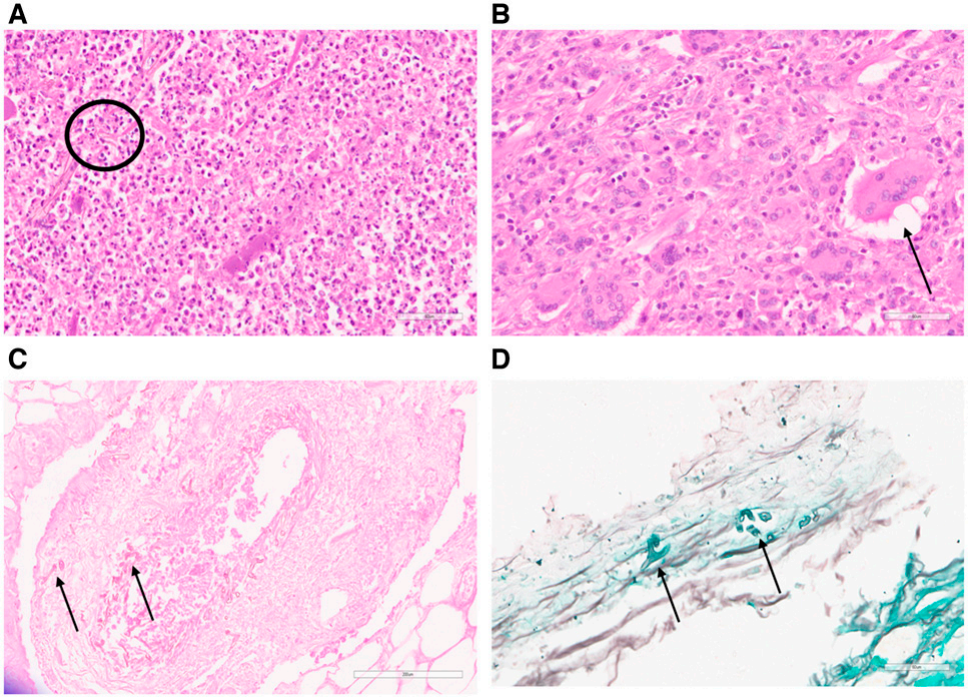
Histopathological assessment of infected rhino-orbital tissues. Orbital tissues with presumed mucormycosis were sectioned to 7-µm thickness and subjected to histopathology analysis. (**A**) Hematoxylin and eosin (H&E) stain of tissue sections demonstrates acute fulminant disease with neutrophilic abscess and necrosed tissue with little aseptate fungus (black circle); 40×. (**B**) H&E-stained tissue sections represent chronic granulomatous disease presented with multinucleated giant cells, epithelioid cells, and chronic inflammatory cells (black arrow); 40×. (**C**) Periodic acid-Schiff stain confirmed the presence of Mucorales (black arrows). (**D**) Grocott-Gomori methenamine silver stain highlights the aseptate broad and wide-angle fungal filaments (black arrows).

Histopathology of ROM presented with acute fulminant and chronic granulomatous inflammation. In acute fulminant disease, tissue biopsy showed neutrophilic abscesses and necrosis (Figure 2A), whereas in chronic granulomatous inflammation, granuloma formation with multinucleated giant cells and chronic inflammatory cells was noted (Figure 2B). This fungus has affinity for the endothelial cells of blood vessels, as it was also found that fungus was arranged over the blood vessel wall and present as vascular emboli inside the lumen of blood vessels. The cell wall of Mucorales gave a magenta color when stained with PAS stain (Figure 2C). Grocott-Gomori methenamine silver stain highlighted the aseptate broad and wide-angle fungal filaments (Figure 2D).

## DISCUSSION

Our study revealed middle-aged (27- to 60-year-old) males to be the most affected and the majority were found to be diabetic at the time of diagnosis of mucormycosis. It was noteworthy that a fair proportion of subjects had a history of hospitalization during the course of COVID-19 illness. Additionally, the majority of our patients had a definite history of receiving steroids, similar to another study from India,^9^ but it was not evident whether the immune dysregulation observed that made them prone to mucormycosis was due to the use of steroids or prior hospitalization. The presence of multiple risk factors could also make individuals more susceptible to ROM because they create an immunosuppressive environment^15^^–^^17^ and, in our study, we considered age, diabetes, COVID-19, and steroids as risk factors associated with mucormycosis.

Whereas all patients were given TRAMB, 10 patients additionally had exenteration of the eye. Early and accurate laboratory diagnosis is the most important factor for improving survival in patients with mucormycosis. In our study, an immediate diagnosis was possible in 12/24 specimens by deep or endonasally guided nasal swabs, depicting the utility of nasal swabs and biopsies to make a quick definitive diagnosis of ROM. Nasal swabs were not collected from the rest of the 12 patients because the infection was severe and had reached orbit. Therefore, samples were obtained after exenteration and orbital debridement. Calcofluor white is a special fluorescent stain that binds strongly to structures containing cellulose and chitin, and CFW stain from a nasal biopsy showing the typical irregular and rare septate hyphae that branch at right angles is characteristic of the Mucorales group.

However, fungal culture is necessary to identify the genus; the Mucorales group is usually fast growing and within 1–2 days would form confluent, raised, fluffy colonies on routine media. However, the recovery of these organisms can be difficult in culture,^18^ due to the fragile nonseptated growth of these fungi that makes them easily mechanically damageable during sample manipulation. The agents of mucormycosis vary depending on the geographical area. Other isolated fungi belong to the genera *Lichtheimia*,* Mucor*,* Rhizomucor*,* Cunninghamella*,* Saksenaea*, *Apophysomyces*,* Cokeromyces*,* Actinomucor*, and *Syncephalastrum*. Globally, *Rhizopus* spp., *Lichtheimia* spp., and *Mucor* spp. account for 75% of all cases.^5^^,^^6^ In India, *Rhizopus oryzae* and *Apophysomyces* spp. are mainly found. Although Mucorales are considered opportunistic pathogens, *Apophysomyces* and *Saksenaea* spp. are responsible for cutaneous mucormycosis in immunocompetent patients, and *R. homothallicus*, which was isolated in our study, had been earlier reported for the first time from patients with cavitary pulmonary mucormycosis.^19^ Interestingly, there was no association of any species with any anatomic infection site and outcome.^20^ A recent study by Gupta et al.^21^ reported that the most common Mucorales found to cause the epidemic was *R. oryzae* followed by *R. microsporus*, which is similar to the findings in our study. Reported worldwide, *R. oryzae* is the most common fungus isolated from clinical specimens of patients with mucormycosis^22^ and, in the current study, the most common fungus was *R. arrhizus* followed by *R. microsporus*. Clinically, it can mimic *Aspergillus* sp., and hence a high index of suspicion is required for early recognition of mucormycosis. One important clue is progressive necrosis despite adequate medical therapy. Even with timely diagnosis, the window of opportunity is much shorter because they grow very rapidly in vivo. Therefore, effective treatment should be initiated before extensive angioinvasion occurs.^23^ Earlier studies suggest ROCM to have the worst outcome and poor prognosis despite antifungal therapy.^5^^,^^9^^,^^22^ In our study, we aimed to elaborate the type of Mucorales isolated in our population post COVID-19 and to determine its antifungal susceptibility because understanding the type of species involved could help us correlate the disease prognosis and severity. However, in our study, we did not find any difference in the disease progression or treatments administered regardless of the fungal species. Gupta et al.^21^ also reported that amphotericin B, posaconazole, and isavuconazole had the lowest MIC values in 98.8% of the Mucorales identified, whereas our study showed that the susceptibility to posaconazole was high, with a MIC value less than 2 µg/mL in 84% of the Mucorales followed by amphotericin B and natamycin. Thus, our study shows that Mucorales are resistant to many antifungals, including ketoconazole, voriconazole, and caspofungin, with variable susceptibility to natamycin. Amphotericin B and posaconazole are the only promising drugs based on antifungal susceptibility testing. Combined medical and surgical intervention has improved survival rate over medical treatment alone.^9^

To conclude, mucormycosis, an insidious killer, should be an important differential diagnosis when broad aseptate filaments are observed by direct microscopy, and molecular tests using 28S rRNA PCR is a good addition to the armamentarium to make a rapid identification of the species involved. With the waves of the COVID-19 pandemic ongoing, a high index of suspicion in certain clinical settings cannot be overemphasized.
